# Musculoskeletal care pathways for adults with hip and knee pain at the interface between primary and secondary care: protocol for a systematic review

**DOI:** 10.1186/s13643-016-0301-x

**Published:** 2016-08-01

**Authors:** Kate Button, Fiona Morgan, Helen Hodgson, Alison L. Weightman, Steve Jones

**Affiliations:** 1 School of Healthcare Sciences, Cardiff University, Eastgate House, Newport Road, Cardiff, CF24 0AB UK; 2 Trauma and Orthopaedics, Cardiff and Vale Orthopaedic Centre, University Hospital Llandough, Cardiff and Vale UHB, Cardiff, UK; 3 Specialist Unit for Review Evidence (SURE), Cardiff University, Cardiff, UK

**Keywords:** Musculoskeletal, Hip pain, Knee pain, Care pathway, Service delivery

## Abstract

**Background:**

Musculoskeletal (MSK) conditions are the most frequently reported chronic conditions and one of the biggest causes of disability in the UK. Given the ageing population and the impact of these problems, the demand for MSK treatment will rise. Despite reduced waiting times, MSK pathways have remained variable and inconsistent and need to be improved to meet patient needs. The aim of this systematic review is to understand the evidence for the effectiveness of current models of service delivery and care pathways for adult hip/knee pain patients accessing secondary care for specialist opinions.

**Methods:**

MEDLINE, MEDLINE In-Process, CINAHL, Embase, PEDro, PubMed, Web of Science, Cochrane Central and HMIC databases will be searched without language restrictions for papers published from 1990 onward. Websites will be reviewed for grey literature including care pathways, policy documents and unpublished MSK research. Additionally, reference lists will be checked and citations tracked for included studies.

**Discussion:**

The following evidence will be included: research considering care pathways at the intersection between primary and secondary care for adults with hip and/or knee pain in countries with an established clinical pathway. Studies considering generalised inflammatory arthropathy and post-surgical care pathways will be excluded. Screening for included data will be conducted independently by two reviewers. After benchmarking, quality assessment and data extraction will be conducted by one reviewer and checked by a second. A mixed method analysis will be conducted.

This systematic review will be used as part of a programme of research to identify best practice for MSK hip and knee pain care pathways. It will provide recommendations for pathway re-design to meet patient needs and ensure efficient streamlining of the patient journey. The review will combine a wide range of information sources including patient and clinician opinion, clinical guidelines, health service delivery research and stakeholder requirements. This should result in a pathway that provides better patient experience and outcomes, whilst meeting the demands placed on the NHS for high-quality evidence-based interventions with efficient use of resources.

**Systematic review registration:**

PROSPERO CRD42016035510

**Electronic supplementary material:**

The online version of this article (doi:10.1186/s13643-016-0301-x) contains supplementary material, which is available to authorized users.

## Introduction

Musculoskeletal (MSK) conditions are common and are one of the biggest causes of disability [1]. In the UK, an estimated 30.6 million working days are lost annually through these disorders [2], and they are the second largest cause of global years lived with disability [1]. Studies have shown that approximately 30 % of consultations with a general practitioner are about MSK complaints [3]. Knee and hip osteoarthritis represents one of the biggest burdens alongside low back problems, impacting on pain, function and quality of life [4, 5]. Given projected increases in the numbers and proportions of older people in the population, the impact of these problems and the demand for MSK treatment are both likely to rise [4].

Current UK treatment guidance advises that patients with osteoarthritis-related hip and knee pain should be managed in primary care, by the appropriate health professional [6]. If a specialist opinion is required, then the patient will be referred for assessment in a triage clinic, at the interface between primary and secondary care. In the triage clinic, a patient may be seen by any one of a range of healthcare professionals who specialise in MSK conditions. The outcome of this consultation could be a care recommendation such as further investigation, imaging, physiotherapy, injections, medical devices or recommendation for surgical management [3, 6]. Despite guidance having been available for several years, it is clear that multiple pathways are being used inconsistently to manage MSK patient care, with a lack of uniformity across different regions [3]. In more general terms, it has also been observed that health services are arranged around structures that are already in place, rather than the needs of the patients receiving the care. This failure to consider the level of need required can result in over- or less effective treatment [7].

Given the rising numbers seeking treatment and the pressure this will place on National Health Service (NHS) resources, it is essential that the care pathway for those experiencing hip and knee pain is based on robust evidence [3]. A recent systematic review evaluating the role of allied health professionals in interface clinics for MSK conditions found that the clinics were well received by patients, were cost effective and did have the potential to improve outcome. However, these findings were based on a low level of evidence [8].

The need to re-design the pathway for MSK conditions, to make sure that the patient sees the right clinician, at the correct stage in their condition management, was highlighted in a Canadian cohort study. The study, which evaluated the consultation outcomes of orthopaedic consultants, found that 79.3 % of the patients assessed did not go on to have surgery within 18 months of the consultation. It concluded that further research was needed on both the best model of care and how to use the scarce and expensive resource of the orthopaedic surgeon most appropriately [9].

When re-designing the pathway, consideration of patient values, beliefs and needs are also very important. The authors in [10], exploring the illness beliefs in patients with knee osteoarthritis, found that many individuals there shared a strong belief that surgery was an unattractive treatment option. This is crucially important given that referrals to specialist care often result in automatic surgical assessment. Findings such as these support the need for a care pathway that begins with patients having the opportunity to explore conservative treatment first.

None of the previous systematic reviews in this field have specifically explored care pathways and evaluated evidence from all the different aspects of healthcare delivery that need to be considered: patient needs, service organisation, outcomes and costs. If the hip and knee pain care pathway is to be re-designed effectively to meet future healthcare needs, it is crucial to have a full picture of the existing evidence.

This systematic review is part of a programme of research for patient and public benefit that is being funded by Health and Care Research Wales. The aim of the review is (1) to understand the evidence for the effectiveness of current models of service delivery and care pathways for adult hip/knee pain patients accessing secondary care for specialist opinions and (2) to identify the key information required for effective referral decisions to ensure the most appropriate intervention for individual patients.

This data will be used to re-design the care pathway for patients with hip and knee pain from the point of referral for specialist care.

## Methods

This systematic review protocol has been designed in accordance with the Preferred Reporting Items for Systematic Review and Meta-Analysis Protocols (PRISMA-P) guidance [11]. The PRISMA-P is included as Additional file 1.

### Search strategy

Searches will be conducted for journal publications and grey literature investigating models of service delivery and care pathways. All sources will be searched without language restrictions for evidence published from 1990 onward. The choice of 1990 as the start date for the search is based on the understanding that care pathways started to be introduced in the early part of the decade [12]. The following databases will be searched: MEDLINE, MEDLINE In-Process, Cumulative Index to Nursing and Allied Health Literature (CINAHL) Embase, PEDro, PubMed, Web of Science, Cochrane Central Register of Controlled Trials and Health Management Information Consortium (HMIC).

Using a range of resources including TerMine [13] to identify keywords and Yale MeSH Analyzer [14] for indexed terms, the search terms have been developed in Ovid MEDLINE (Table 1) and will be adapted to the other databases. The Ovid MEDLINE strategy was checked, using a set of relevant papers, to ensure a reasonable balance of sensitivity (ability to pick up known relevant studies) and precision (number of papers needed to screen per relevant paper). It is notable that the terms include triage as both a keyword and an indexed term in two sections of the strategy. This is deliberate because they are used (i) to describe the decisions taken within a care pathway and (ii) specifically to identify a move from primary to secondary care.

**Table 1:** Search terms: Ovid MEDLINE

MEDLINE	
#	Searches
1	musculoskeletal.ti,ab.
2	hip.ti,ab.
3	knee.ti,ab.
4	orthopaedic.ti,ab.
5	orthopedic.ti,ab.
6	musculoskeletal diseases/
7	musculoskeletal pain/
8	or/1-7
9	(Care adj3 (map* or pathway* or protocol* or access*)).ti,ab.
10	(clinic* adj3 (standard* or pathway* or assessment* or map* or protocol*)).ti,ab.
11	((critical or patient*) adj3 pathway).ti,ab.
12	(screening adj3 (service* or standard* or protocol*)).ti,ab.
13	triage.ti,ab.
14	critical pathways/
15	triage/
16	or/9-15
17	triage.ti,ab.
18	MCAT.ti,ab.
19	MCASS.ti,ab.
20	conversion rate.ti,ab.
21	(extended scope adj3 physiotherap*).ti,ab.
22	(orthop?edic adj (consultation or surgeon)).ti,ab.
23	MCAS.ti,ab.
24	CATS.ti,ab.
25	(primary adj2 secondary).ti,ab.
26	primary-secondary.ti,ab.
27	(refer adj6 (GP or general practi* or primary care or outpatient or consultation or nurse practitioner*)).ti,ab.
28	ambulatory care/or ambulatory care facilities/
29	“referral and consultation”/
30	triage/
31	or/17-30
32	8 and 16 and 31
33	limit 32 to yr=1990-2016
34	exp fractures, bone/
35	exp emergency services, hospital/
36	(fracture* or emergency).ti.
37	or/34-36
38	33 not 37

In addition to searching databases, a range of supplementary techniques will be employed. The following websites will be searched for grey literature, care pathways and policy documents using keywords and potentially relevant results identified:

AHRQ National Guidelines Clearinghouse http://www.guideline.gov/

Arthritis Australia www.arthritisaustralia.com.au/

Arthritis Care UK http://www.arthritiscare.org.uk/

Arthritis Foundation www.arthritis.org

Arthritis National Research Foundation http://www.curearthritis.org/

Arthritis New Zealand www.arthritis.org.nz/

Arthritis Research UK http://www.arthritisresearchuk.org/

Arthritis Society www.arthritis.ca/

European Pathway Association http://www.e-p-a.org/

Open Clinical http://www.openclinical.org/clinicalpathways.html

OpenGrey http://www.opengrey.eu/

NICE Evidence Search https://www.evidence.nhs.uk/

TRIP database https://www.tripdatabase.com/

Finally, for included papers and documents, reference lists will be checked, citations tracked and authors contacted to identify additional evidence.

A log of all searches will be kept with details of search terms used for each database or website. The results will be exported to an electronic database (EndNote version X7.1, Thomson Reuters, New York) for storage and management of all identified citations.

### Eligibility

The review will consider all study designs and documents that look at care pathways for MSK hip and/or knee pain for adults aged 18 and over accessing secondary care for specialist opinion. A MSK pathway generally accepts all hip/knee pain without requiring specific diagnostic criteria. The European Pathway Association defines a care pathway as “a methodology for the mutual decision making and organization of care for a well-defined group of patients for a well-defined period” [15].

Publications looking at the following will be excluded: generalised inflammatory arthropathy, stroke and MSK care pathways that do not include the hip and knee joint or which focus on post-surgical care.

### Data selection

After duplicate results are removed, titles and abstracts will be reviewed independently by two reviewers to determine whether they meet the eligibility criteria. All papers and documents potentially meeting the criteria will be obtained in full text and reviewed for inclusion by two independent reviewers. At both the title/abstract and full-text stages, cases of disagreement will be resolved by discussion with a third reviewer.

The data selection process will be documented using the PRISMA flow diagram [16]. A record will be kept of all items excluded at full text with the reason for their exclusion.

### Quality assessment

Quality assessment will be conducted using validated checklists for specific research designs (e.g. randomised controlled trials, qualitative studies, cross-sectional surveys, longitudinal studies, case series). The forms will be pilot tested to ensure a baseline of understanding and agreement between reviewers is achieved. Following this, studies will be assessed by one reviewer with that assessment being checked by a second reviewer.

### Data extraction

Evidence will be extracted directly into an agreed form. Template forms for different study designs have been developed by team members and used in previous research. These forms will be adapted and agreed to ensure all appropriate data are collected. As with quality assessment, the form will be pilot tested on a sample set of studies to ensure a baseline of understanding and agreement between reviewers is achieved. After piloting, each data extraction form will be completed by one reviewer and checked for accuracy by another.

It is anticipated that data relating to the following outcomes will be extracted: patient experience, expectations, illness beliefs and satisfaction, consultation outcomes (numbers/resources triaged), costs and utilisation of workforce and healthcare resources. If additional outcomes are identified, data for these outcomes will also be extracted. Given the scope of the review, no primary outcome has been defined.

### Data synthesis

Data synthesis will be conducted in three phases: effectiveness, participant views and a cross-study synthesis as outlined by Kavanagh et al. [17].

#### Effectiveness

If appropriate to do so (judged qualitatively on the basis of similarity of study design, population included, intervention applied and outcomes measured), meta-analysis will be conducted using the Cochrane Collaboration’s Review Manager. A random effect model will be utilised. Where outcomes are comparable between studies, measures for continuous data will be presented as mean differences (or standardised mean differences as appropriate), 95 % confidence intervals (CIs) and *p* values. For dichotomous data, results will be presented as relative risks with 95 % CIs and *p* values. Meta-analysis results will be presented using forest plots, with embedded risk of bias traffic light tables. Heterogeneity will be quantified and reported using the *I* ^2^ statistic. If more than 10 studies are identified for meta-analysis, publication bias will be visually explored using a funnel plot.

Attrition rates will be recorded for included studies, and if necessary, a sensitivity analysis will be conducted to consider the impact of including studies with substantial levels of missing data (attrition rates greater than 20 %). As far as possible, analyses will be based on intention to treat with all participants analysed in their allocated groups.

Intervention papers will coded for outcomes and demographic data using NVivo 10 software (QSR International, Doncaster, Australia).

#### Participant views

A broad evidence synthesis of view studies (qualitative, correlation, cross-sectional surveys and process evaluations) will be undertaken. Where applicable, data gathered from quantitative studies will be analysed thematically and integrated with the key findings from qualitative studies. Data synthesis will be dependent on the nature of the evidence available. If the body of evidence is sparse and does not share common interventions or themes, a narrative description of the themes in each paper will be presented. Where the evidence is sufficiently rich and shares common themes, a thematic synthesis will be performed.

An index ladder of codes will be developed a priori, in accordance with Richie and Spencer [18], so that key findings can be extracted and organised at the same time. The index ladder of codes will be developed after reading a sample of eligible papers and discussion with the team. The coded findings will be read and re-read by at least two members of the team, and categories may be further refined and organised. The coding framework may also be modified during coding as new themes emerge. Themes will be coded in NVivo by one reviewer and checked by another.

An aggregative rather than an interpretive approach to synthesis will be used [19] bringing together and reflecting the nature of evidence identified, but not developing theoretical concepts.

#### Cross-study synthesis

The final synthesis will juxtapose the findings of the two syntheses against one another to identify interventions and solutions that answer the needs of the target population. This will be presented as a table or matrix dependent on the findings.

### Registration

The protocol for the systematic review and meta-analysis has been registered with PROSPERO, the international prospective register of systematic reviews (PROSPERO 2015: CRD42016035510), available from http://www.crd.york.ac.uk/PROSPERO/display_record.asp?ID=CRD42016035510.

### Protocol amendments

Important variations from the published protocol will be reported in the systematic review.

## Discussion

This systematic review will provide a full picture of the current evidence considering care pathways for hip and/or knee pain at the interface between primary and secondary care. That picture will highlight evidence about which aspects of the pathway work well, what is not working well and key areas for future research. To date, no systematic review has provided a comprehensive analysis that synthesises service delivery and organisation with service user requirements and opinion.

This review is part of a programme of research that is being funded by Health and Care Research Wales. The results will provide data for effective care pathway re-design, by identifying the best model of care. The work will be enhanced by development of a decision aid to support information exchange and efficient referral of patients across the pathway. To achieve this, the findings of the systematic review will be combined with data from service user experience and the key information required to inform referral decisions for these patient groups.

Ultimately, this should result in a pathway that provides better patient experience and outcomes, whilst meeting the demands placed on the NHS for high-quality evidence-based interventions with efficient use of resources.

## Abbreviations

CINAHL, Cumulative Index to Nursing and Allied Health Literature; MeSH, Medical Subject Headings; MSK, musculoskeletal; NHS, National Health Service; PRISMA-P, Preferred Reporting Items for Systematic review and Meta-Analysis Protocols

## Additional file

Additional file 1: PRISMA reporting checklist. (DOC 107 kb)
